# The Treatment of Chronic Renal Disease

**Published:** 1906-03-10

**Authors:** 


### THE TREATMENT OF CHRONIC RENAL DISEASE.

In a paper on this subject read before the Medical Society of London Dr. Samuel West considered principally points on which modern opinion is undecided or conflicting. Dealing first with chronic parenchymatous nephritis, the writer said the problem was how to promote the growth of the renal cells. To secure this end effective nutrition was necessary, and this was impossible if a too prolonged course of purely milk diet was practised. Meat extracts and alcohol should always be excluded, but this restriction did not apply to eggs in moderation, and there was no sufficient reason why white meat should be ordered and red meat always forbidden. Similarly, confinement to bed or to one room was often too long persisted in. Under a more liberal diet and open-air treatment (short of exposure to chill) rapid improvement often took place. In granular kidney the treatment must differ in each of the three stages of the malady. In the earliest stage much might be done to retard the progress of the disease. Over-exertion and exposure should be avoided, but moderate, regular exercise was desirable. In the middle stage of cardiac hypertrophy and thickened arteries treatment should be directed to prevent the risks of cardiac failure, rupture of vessels, and intercurrent nephritis. Hence the necessity of avoiding strain, whether physical or mental. If arterial tension became subnormal, digitalis might be necessary, but otherwise, though useful for the heart, it increased peripheral resistance by contracting the arterioles. On the other hand caffeine acted as a cardiac stimulant without contracting the vessels. In the later stages of the disease the symptoms became more pronounced, and might arise in almost any part of the body. Arterial tension now varied greatly. If low, it must be raised by diet, stimulants, digitalis, ergot, and adrenalin. High tension, on the other hand, demanded modification of the diet in the opposite direction; also purgation, baths, diaphoretics, nitro-glycerine, and potassium iodide. Pilocarpine is a most useful drug, and its use is free from the risks sometimes said to attend it; it is particularly valuable for restlessness, sleeplessness, and threatening uræmia. As a hypnotic cannabis indica is of service. In some cases the administration of renal extract seemed to do good.

In the discussion which followed Dr. West's paper Sir Lauder Brunton advocated the use of strophanthus in the middle stage of the disease as this drug acted as a cardiac tonic without constricting the blood-vessels. With high arterial tension 20 grains of potassium nitrate and 1 grain of sodium nitrite in a tumblerful of water every morning was useful; it lowered the tension, and might without harm be continued for years. Dr. Haviland Hall considered that milk diet should always have a fair trial—three to four weeks—in chronic nephritis. He also emphasised the value of pilocarpine. In granular kidney he used strophanthus in combination with potassium iodide. Headache associated with high tension would frequently be relieved by one-third of a minim of solution of trinitrin, frequently repeated if necessary.

